# Role of Thioredoxin-Interacting Protein in Diseases and Its Therapeutic Outlook

**DOI:** 10.3390/ijms22052754

**Published:** 2021-03-09

**Authors:** Naila Qayyum, Muhammad Haseeb, Moon Suk Kim, Sangdun Choi

**Affiliations:** 1 Department of Molecular Science and Technology, Ajou University, Suwon 16499, Korea; nailaqayyum16@gmail.com (N.Q.); haseeb3389@hotmail.com (M.H.); moonskim@ajou.ac.kr (M.S.K.); 2 S&K Therapeutics, Woncheon Hall 135, Ajou University, Suwon 16499, Korea

**Keywords:** thioredoxin, TXNIP, metabolic disorders, neurological disorders, TXNIP modulator

## Abstract

Thioredoxin-interacting protein (TXNIP), widely known as thioredoxin-binding protein 2 (TBP2), is a major binding mediator in the thioredoxin (TXN) antioxidant system, which involves a reduction-oxidation (redox) signaling complex and is pivotal for the pathophysiology of some diseases. TXNIP increases reactive oxygen species production and oxidative stress and thereby contributes to apoptosis. Recent studies indicate an evolving role of TXNIP in the pathogenesis of complex diseases such as metabolic disorders, neurological disorders, and inflammatory illnesses. In addition, TXNIP has gained significant attention due to its wide range of functions in energy metabolism, insulin sensitivity, improved insulin secretion, and also in the regulation of glucose and tumor suppressor activities in various cancers. This review aims to highlight the roles of TXNIP in the field of diabetology, neurodegenerative diseases, and inflammation. TXNIP is found to be a promising novel therapeutic target in the current review, not only in the aforementioned diseases but also in prolonged microvascular and macrovascular diseases. Therefore, TXNIP inhibitors hold promise for preventing the growing incidence of complications in relevant diseases.

## 1. Introduction

Thioredoxin-interacting protein (TXNIP) was first identified in cancer cells as a vitamin D3 target gene and later known as vitamin D3 upregulated protein 1 (VDUP1). Its activity or expression is regulated at metabolically essential sites, such as liver cells, adipose tissues, and skeletal muscle, while it is most abundantly expressed in the glomeruli of human and rat kidneys [1]. TXNIP belongs to the α-arrestin protein family; these scaffolding intermediary proteins play key roles in multiple signaling pathways. TXNIP interacts directly with two cysteine (Cys) residues at the active catalytic site of reduced thioredoxin (TXN), further blocking its potential for scavenging reactive oxygen species (ROS). The interaction between reduced TXN and TXNIP through a disulfide linkage is essential for their basic protein–protein interaction. TXNIP further modulates TXN’s protein structure while reorganizing de novo disulfide bond synthesis on Cys, with unique residues at positions 32 and 247, respectively [2,3].

Several antioxidants, such as thioredoxin, glutaredoxin, and glutathione, help to maintain the activity of the TXN-system via a cell-based redox (reduction/oxidation) mechanism, which could face direct oxidative stress if ROS production is dysregulated [4]. The TXN system is an important regulator for the maintenance of a cellular reduced environment involving nicotinamide adenine dinucleotide phosphate (NADPH), TXN reductase, and TXNIP. TXNIP interacts with TXN and activates it as a negative regulator, which directly affects the redox balance [5]. TXNIP is involved in maintaining cell integrity by participating not only in proliferation, differentiation, autophagy, pyroptosis, inflammation, and apoptosis but also modulating gene expression, metabolism, and redox reactions [6,7,8,9]. Several studies have revealed upregulation of TXNIP in diseases like type 2 diabetes mellitus (T2DM) [8], type 1 diabetes mellitus (T1DM) [10], cardiovascular diseases [11], ischemic stroke, and cataract [9] as well as neurodegenerative disorders such as Alzheimer’s disease (AD), and Parkinson’s disease (PD) [12,13]. Conversely, other studies have emphasized reduced TXNIP expression in tumor cells [14], mostly solid cancers [15,16]. Recent research has indicated its regulation via mechanistic controls such as heat shock and hypoxic conditions as well as biochemical controls, such as those mediated by H_2_O_2_, NO, insulin, and glucose [17,18,19,20,21]. In the past decade, TXNIP has emerged as an essential metabolic regulator of lipid and glucose metabolism [22]. In addition, it modulates the transcription of several genes, each of which points to new mechanisms implicating TXNIP as a therapeutic target in several disorders [23,24,25,26]. Lastly, regarding the aforementioned diseases, we would briefly mention TXNIP participation in the pathogenesis of diabetic complications and intensively studied neurodegenerative diseases as well as the emerging interest in the development of new therapeutic approaches. This review is aimed at highlighting the roles of TXNIP in the field of diabetology, neurodegenerative diseases, and inflammation.

## 2. Signaling Pathways Involving Thioredoxin-Interacting Protein (TXNIP)

### 2.1. The Inflammatory Pathway

TXNIP has been reported to play a vital part in diabetes during the immune response by activating the inflammatory pathway via the NLRP3 (NOD-, LRR-, and pyrin domain–containing protein 3) inflammasome. The physical association between TXNIP and NLRP3 is a part of the NLRP3 inflammasome multiprotein complex (consisting of an adaptor protein, apoptosis-associated speck-like protein containing a caspase recruitment domain (ASC), cardinal, and caspase 1) [27,28]. Recently, high glucose concentrations were found to promote the activation of the NLRP3 inflammasome [29,30]. Another mechanism induces TXNIP by endoplasmic-reticulum stress (ERS; once the capacity of the ER to fold proteins reaches a saturated level); misfolded proteins induce the cell to consume reduced TXN to obtain energy in the form of NADPH for modifying these proteins (for correction), thereby causing cellular stress that triggers an inflammatory response. The upregulation of TXNIP by ERS is mediated by inositol-requiring enzyme 1a (IRE1a) and protein kinase R-like endoplasmic reticulum kinase (PERK) in β-cells [31]. IRE1a overexpression activates TXNIP via reducing activity of microRNA-17 (miR-17), which is a TXNIP-destabilizing microRNA. The transcriptional expression of *TXNIP* may be boosted by carbohydrate response element–binding protein (ChREBP) nuclear translocation and activating transcription factor 5 (ATF5, an ATF/cAMP response element) [32,33,34]. Meanwhile, it is suggested that in pancreatic β-cells, forkhead box O1 (FOXO1) binds to the ChREBP/*TXNIP* promoter region and acts as a TXNIP suppressor [35]. Elevated expression of IRE1a and PERK-eIF2a results in transcription and overexpression of *TXNIP*, which ultimately activates the NLRP3 inflammasome. Nod-like receptors (NLRs) sense endogenous cell signals in the form of stress, damage, or abnormal death, whereas exogenous signals are associated with pathogens [36,37]. At present, all known NLRP3 activators are believed to induce ROS production, while inhibitors of ROS also block the NLRP3 inflammasome [38]. Although there are several ways to activate the NLRP3 inflammasome, upstream ROS activation by multiple factors acts on the TXNIP–TXN key complex and dissociates it. The liberated TXNIP, therefore, activates the NLRP3 inflammasome and ultimately caspase 1 to stimulate the release and maturation of interleukin 1β (IL-1β) and IL-18. Further elucidation of this phenomenon suggests maintenance of the macrophage and β-cell activation in an autocrine and paracrine manner, thereby intensifying the inflammatory responses [30]. Pyroptosis is another type of programmed cell death associated with inflammatory caspase 1 and proinflammatory regulators that are common in necrosis and apoptosis [27], and is closely linked to the activation of the inflammatory pathway. As observed in ERS involving the TXNIP/NLRP3 cascade with elevation in the degradation of miR-200a induced pyroptosis and leads to renal failure in a mouse model [31].

### 2.2. A Metabolic Pathway

**Figure 1:**
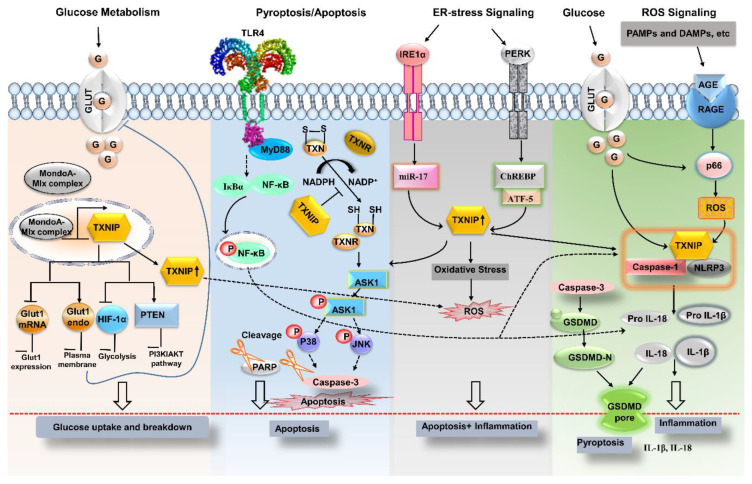
Upstream mediators of TXNIP signaling. Possible signaling mechanisms by which TXNIP launches abnormal signaling resulting in disease symptoms may include the following. In glucose metabolism, the elevation in glucose uptake correlates with an increase in mRNA expression of *Glut1*, which is supported by TXNIP regulation; the blue line indicates the modulation of glucose metabolism via inhibition of its breakdown and uptake; TLR4 action mediated by MyD88 activates the NF-κB pathway, where it interacts with the main intermediaries of inflammation in a TXNIP/NLRP3-associated manner and alleviates pyroptosis; upregulated oxidative stress and multiple upstream intermediaries upregulate ROS, which further elevates TXNIP expression, thereby disrupting the interaction of TXN and ASK1. Hence, liberated ASK1 upon interaction with caspase 3 leads to generic apoptosis; the ERS-mediated TXNIP-associated inflammasome pathway drives apoptosis and inflammation with an eventual release of cytokines (IL-1β and IL-18). Inflammatory-pathway upstream inducers include ROS, glucose, and ERS. ERS: endoplasmic-reticulum stress; PERK: protein kinase RNA-like ER kinase; IRE1α: inositol-requiring enzyme 1α; eIF-2α: eukaryotic translation initiation factor 2α; TLR4: toll-like receptor 4; TXN: thioredoxin; TXNR: thioredoxin reductase; TXNIP: thioredoxin-interacting protein; NLRP3 inflammasome: Nod-like receptor protein 3 inflammasome; IL-1β: interleukin 1β; ATF5: activating transcription factor 5; ChREBP: carbohydrate response element–binding protein; ROS: reactive oxygen species; ASK1: apoptosis signal-regulating kinase; Overexpression(↑); Inhibition(⊣); Involving intermediaries (⇢).

TXNIP influences metabolic pathways as a key regulator of lipid and glucose metabolism, including multiple actions via glucose uptake from peripheral tissues such as muscles and adipose tissue as well as glucose production in the liver. Nonetheless, TXNIP expression is reciprocally associated with glucose transporter 1 (Glut1) in prostate cancer, whereas in diabetes, it also activates the negative-feedback loop to regulate glucose assimilation in response to a rise in glucose concentration [12,14]. Studies have also suggested that TXNIP can modulate the expression and position of Glut1 to prevent glucose uptake. Direct transcription control is evident in HepG3 cells, where the loss of TXNIP is associated with significantly elevated expression of *Glut1* and high uptake of glucose. Further studies have revealed that TXNIP acts directly on Glut1 in the plasma membrane and reduces its protein amount by inducing endocytosis [39,40] (Figure 1). Theoretically, TXNIP may increase mitochondrial oxygen consumption by hindering the function of hypoxia-induced transcription factors (HIFs) or may induce peroxisome proliferator-activated receptors, indeed TXNIP as a shuttling protein interact with HIF-1α and translocates it from nucleus to cytosol for degradation [41]. HIF-1α inhibits the activity of pyruvate dehydrogenase by hampering the tricarboxylic acid cycle (TCA) cycle, ultimately leading to a decrease in ROS levels. Conversely, the mechanism through which TXNIP regulates the TCA cycle remains elusive [42]. Furthermore, TXNIP regulates and activates phosphatase and tensin homolog (PTEN) lipid phosphatase in a redox-dependent manner [43]. By contrast, PTEN inhibits the AKT–PI3 kinase pathway that further downregulates glucose uptake and metabolism [44]. The cost of TXNIP loss–related mitochondrial oxidative damage is associated with increased release of NADP(H)/NADH and greater blockage of reductive restimulation of PTEN, with enhanced activation of PI3K–AKT signaling and boosted metabolism and glucose transport [43,45]. Broadly speaking TXNIP plays an important role in metabolic regulation, partially independent of its ability to bind to TXN [8].

### 2.3. The Apoptotic Pathway

It is suggested that TXNIP is involved as a pro-apoptotic protein in β-cells, likewise widely expressed in ischemic diseases [46]. It plays an inhibitory role in the activity of the TXN system, which is pivotal for maintaining the optimally reduced cellular environment [47]. In the cytoplasm, TXNIP inhibits TXN action in a redox-dependent manner by binding to TXN and relocalizing from binding of protein competitively or redox independently, via an increase in TXNIP stability due to high glucose induction [4] (Figure 1). The proapoptotic characteristics of TXNIP have been reported in several types of brain injury and in in vitro microglial thrombin-associated models [48,49]. Supportive evidence suggests that genetic knockdown of TXNIP exhibits positive effects on mitochondrial functions and is associated with modulation of the mitochondrial death pathway via glucotoxicity-induced apoptosis [2]. Notably, elevated TXNIP expression has been documented in apoptosis and accelerated early brain injury (EBI) following the subarachnoid hemorrhage (SAH) [50]. In human macrophages, TXNIP mitochondrial translocation has been demonstrated during increased ROS production and NLRP3 inflammasome activation [51,52]. These findings imply that TXNIP silencing acts as a therapeutic response to other antioxidants such as quercetin and ascorbic acid and are significant in relation to the development of treatments of diabetic retinopathy (DR). In mitochondria, it binds to TXN2 and hinders the inhibitory association of TXN2 with ASK1; then, the liberated ASK1 is phosphorylated, initiating cytochrome c release, and caspase-3 activation, consequently leading to the activation of ASK1 and apoptotic-kinase pathways [2,53]. Recent findings have shown that modulation of the mitochondrial apoptotic cascade might promote a pathology of the central auditory cortex (central presbycusis) [54]. A parallel study demonstrated that in tempered prediabetic neuropathy in a dyslipidemia mouse model, inhibition of TXNIP-facilitated apoptosis and inflammation can lead to neuronal loss in the dorsal root ganglion [55]. In the nucleus, TXNIP induces β-cell apoptosis by increasing the expression of proapoptotic miR-204 and miR-200. Moreover, TXNIP-related apoptosis studies in oncology are also underway [56]. Furthermore, microRNAs can decrease the expression of relevant target genes, including musculoaponeurotic fibrosarcoma oncogene homolog A (MafaA), which induces insulin reduction and damages β-cell function [57].

## 3. The Role of TXNIP in Diseases

### 3.1. TXNIP in Diabetology

Diabetes mellitus (DM) is a metabolic disorder regulated by a glucose-lowering hormone known as insulin produced by pancreatic β-cells; the release of insulin is not adequate, which results in DM [58,59]. The anomalous reaction of target tissues to insulin-mediated effects, combined with glucose production-promoting hormone glucagon, may enhance aberrant gluconeogenesis leading to hyperglycemic conditions, which predispose to T2DM [60,61]. TXNIP is a prominent regulator of glucose homeostasis through regulating gluconeogenesis in the liver and is implicated in adaptation to acidosis with ATP generation [62]. Although chronic hyperglycemic conditions promote several metabolic vascular complications associated with high death rates in diabetic patients [63], they may include an increase in the formation of advanced glycation end products (AGEs) and ROS [64,65] (Figure 1). Diabetic models show that ROS are not the only factor that promotes DM, but the overall activity of the antioxidant system may be disrupted in DM [66]. TXNIP deletion appears to be pro-oxidant, and reported to lessen the ROS production in vascular smooth muscle cells indirectly implying an increase in the antioxidant potential of TXN in vitro [67]. Moreover, in mouse models of glucose-induced DM, glucose enhances TXNIP expression, which can further induce excessive ROS production in the mitochondria and cytosol. TXNIP is an endogenous inhibitor of the main antioxidant mechanism, i.e., the TXN system, and hyperglycemic conditions have been shown to play a key role in vascular diabetic complications. Upregulated TXNIP is observed in peripheral blood and cultured cells from a diabetic mouse model as well as in pancreatic islets of DM patients [68]. Additionally, TXNIP is important for the promotion of angiogenesis because TXNIP activates and regulates the main angiogenic cytokine known as vascular endothelial growth factor (VEGF). TXNIP overexpression in diabetes regulates the activity of the key cytokine VEGF in a glucose-sensitive manner, whereas a TXNIP knockdown by small interfering RNA (siRNA) can overcome the diabetes-related pathologies of angiogenesis and arteriogenesis and may help to recover an ischemic hindlimb [69]. Moreover, supporting action on islet biology was concurrently revealed in another study through reversion of impaired endothelial cell angiogenic function, generation of VEGF, and sensitivity to VEGF activities [26,64]. Recently, TXNIP-knockdown has shown improved anti-senescence and anti-inflammation effects on H9c2 cardiomyocytes under doxorubicin-associated cardiomyopathy [70].

**Figure 2:**
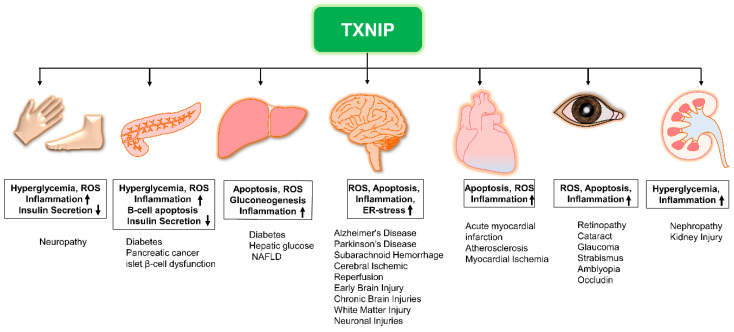
The role of TXNIP in the development of various diseases. Elevated TXNIP expression may lead to the development of various diseases while contributing to these pathologies via distinct mechanisms. NAFLD: non-alcoholic fatty liver disease; ROS: reactive oxygen species; TXNIP: thioredoxin-interacting protein; Upregulation (↑); Downregulation (↓).

Vascular abnormalities in diabetic patients may be attributed to chronic inflammatory responses caused by NLRP3 inflammasome activation. TXNIP also stimulates early apoptotic signals by interacting with inflammation marker, vascular cell adhesion molecule 1 (VCAM-1) in human aortic endothelial cells (HAECs) induced by high-glucose or overexpression of ChREBP, a major transcriptional activator of TXNIP, and impairs nitric oxide (NO) bioactivity; whereas, finally, exaggerated levels of NOs suppress NLRP3 inflammasome activity [64,65,71,72]. Moreover, pyroptosis which is also integrated to the NLRP3 inflammasome activation is associated with diabetes, hypertension, and hyperlipidemia [73]. ERS can control pyroptosis in an alliance of TXNIP with NLRP3 [29]. The literature provides remarkable evidence of elevated ROS and TXNIP levels in diabetic-condition induced NLRP3 inflammasome activation and successive release of caspase 1, IL-1β, and IL-18 (Figure 1). Thus, ROS–TXNIP–NLRP3 inflammasome signaling has a mechanistic link with vascular aberrations in diabetic conditions. The NLRP3 inflammasome directs the obesity-associated danger signal, giving rise to obesity-induced inflammation and insulin resistance. Nevertheless, inhibition of NLRP3 in a mouse model protects against obesity-induced inflammasome activation in the fat-associated pits and liver, and improves insulin signaling [74]. Remarkably, NLRP3 and TXNIP knockout mice show improved glucose tolerance and insulin sensitivity in a T2DM model [29]. Nonetheless, diabetes complications include several complex pathologies, such as nephropathy, retinopathy, neuropathy, ischemic heart disease, peripheral vascular disease, and cerebrovascular disease (macrovascular) (Figure 2).

#### 3.1.1. Diabetic Nephropathy (DN)

Diabetic nephropathy is the most common cause of renal disease and is one of the microvascular complications of DM. Patients show associated symptoms such as proteinuria, abnormal blood hemodynamics, glomerulosclerosis, and thickening of the glomerular basement membrane, which is further protected by podocytes and endothelial cells [75,76]. Accumulating evidence suggests that inflammation is a major factor in the pathogenesis of DN [77,78,79]. The primary mechanism of inflammation control is mediated by the upregulation of ROS, which is in turn controlled by the activation of the nuclear factor-κB (NF-κB) pathway and mitogen-activated protein kinase (MAPK) pathway. In addition, ROS act on the TXNIP–TXN complex, thereby causing it to dissociate, and the dissociated TXNIP functions as a ligand that binds and further activates the NLRP3 inflammasome canonically [80]. The importance of the mitochondrial ROS–NLRP3 inflammasome mediated pathway in DN has been inferred from a knockout mouse model [81]. Recently, in vitro and in vivo studies of glucose-induced TXNIP’s effects on podocyte apoptosis in a DN mouse model suggested that TXNIP deficiency may reduce podocyte apoptosis by inhibiting mammalian target of rapamycin (mTOR) or MAPK signaling cascades [82]. TXNIP deficiency is characterized by attenuated renal injury in diabetic mice, which means that TXNIP could act as a therapeutic target in DN [82,83].

#### 3.1.2. Diabetic Retinopathy (DR)

In diabetic conditions, high-glucose–induced overexpression of TXNIP leads to early apoptosis of neurons, glial activation, and epithelial retinal pigment injury [84]. Recent in vivo studies showed that in retinal microvascular endothelial cells, inhibition of the ROS-induced TXNIP/NLRP3 cascade by vitamin D3 exerts protective effects against anomalies of retinal structure [85]. Therefore, inhibition of ROS-induced TXNIP production in diabetic mouse models can alleviate the apoptosis of retinal cells just as in DN [82,84].

#### 3.1.3. Diabetic Neuropathy

A serious complication of DM, unfortunately poorly studied to date, is characterized by inflammation and associated with sensation loss in peripheral parts of the body or numbness in extremities, such as feet, and is closely associated with TXNIP [86]. The literature supports the idea that TXNIP/NLRP3-mediated signaling leads to IL-1β and IL-18 activation, resulting in canonical inflammation and worsening of diabetic pathogenesis. In contrast, inhibition of this cascade reduces the apoptosis of neurons and delays neuropathic symptoms in prediabetic patients [29]. Recently, it was demonstrated that NF-κB is a crucial regulator of histone deacetylase 2 (HDAC2) and is involved in neuropathic pain through downstream activation of the TXNIP/NLRP3 inflammasome [87,88]. Furthermore, overexpression of miR-23a in spinal glial cells and miR-183 in microglia has been proposed to relieve neuropathic pain in peripheral body parts [86,89]. Thus, TXNIP might affect diabetic neuropathy by amalgamating inflammation and oxidative stress.

### 3.2. TXNIP in Neurology

Neurological disorders such as dementia, AD, PD, SAH, and stroke are the most serious diseases of the modern era. Although there are distinct clinical insights into these pathologies, extensive literature suggests that oxidative stress, mitochondrial damage, inflammation, and dysregulated calcium control contribute to the above diseases [12,13,48,90,91,92]. TXNIP is known to link cellular redox events, mitochondrial redox events, and ERS regulation to pathological inflammation and apoptosis in brain diseases. It also acts as a key mediator in neurodegenerative diseases such as AD and PD [93,94] (Figure 2).

#### 3.2.1. Ischemic/Reperfusion Injury

Ischemic stroke injury is characterized by a blockage in the blood supply to the brain, thereby resulting in sustained deprivation of oxygen supply and leading to brain cell death and damage [95]. TXNIP is overexpressed in ischemic-stroke–induced blood–brain barrier dysfunction and myocardial ischemia/reperfusion injuries [91,96]. TXNIP causes a redox imbalance and leads to inflammasome activation, whereas TXNIP inhibition is an endogenous inhibitor of the thioredoxin system, which helps to reverse ischemic injuries [48]. It has been shown that hypoxic conditions in the ischemic pancreatic cancerous tissue affect the promoter of *TXNIP* and, thus, its transcriptional upregulation, which is equally influenced by HIF-1α [97,98]. Additionally, TXNIP regulates mitochondrial bioenergetics via HIF-1α (an essential regulator of ischemia) modulation in hindering, and peroxisome proliferator-activated receptor 1α (PPAR-1α), as upregulating mitochondrial oxygen consumption [99,100,101]. Nevertheless, the shuttling of cytosolic TXNIP and re-recruitment to mitochondria activates ASK-1, leading to cell death [102]. In the hippocampus, ERS-induced TXNIP/NLRP3-inflammasome activation leads to ischemic neurotoxicity [103]. Moreover, a knockout of *TXNIP* and pharmacological inhibition of TXNIP are reported to protect against brain infarction and neurological diseases in mouse models [104]. So far, the idea to inhibit TXNIP has been elaborated in terms of brain hemorrhage or ischemic stroke, where this protein could serve as a therapeutic target.

#### 3.2.2. TXNIP in Subarachnoid Hemorrhage (SAH)

SAH is a cerebrovascular neurological fatal disorder that reduces brain perfusion and causes bleeding in the space between the brain and the adjacent membrane (subarachnoid space); the major cause of SAH morbidity is early brain injury (EBI) [102]. Elevated levels of *TXNIP* mRNA expression are observed in the patients’ brain samples. Furthermore, a rabbit SAH model has been devised, which features elevated TXNIP levels and decreased TXN reductase expression [105]. Concurrent studies have shown that the inhibition of TXNIP via siRNA suppresses apoptosis and alleviates EBI [102]. Recent studies have suggested that ERS induced via PERK and after downstream development of SAH, can initiate EBI by influencing apoptosis [50]. Further research revealed that TXNIP links ERS with neuronal apoptosis, which in turn intensifies EBI [102]. TXNIP interconnects oxidative stress and neuroinflammation to SAH and EBI; as supporting evidence, apelin-13/apelin receptor (APJ) was recently used to reduce EBI via suppressing ERS-associated TXNIP/NLRP3 inflammasome activation and AMP-dependent-protein kinase (AMPK)-dependent oxidative stress following SAH in rats [106]. Furthermore, the white matter injury occurring at the early stage of SAH has not been addressed well so far. Recently, the damage caused by the SAH peroxisome in mouse models was found to escalate white matter injury to SAH, and was partially mediated by TXNIP and glycerone-phosphate acyl-transferase pathways [107].

#### 3.2.3. Alzheimer’s Disease (AD)

The involvement of TXNIP in AD is mostly associated with inflammation; accumulated data indicate overexpression of TXNIP in the brain via amyloid-β (Aβ) exposure [108,109], and also TXNIP remained an exclusive marker in microglia, neurons, astrocytes, and endothelial cells [110]. The prevalent idea proposes that TXNIP is an essential mediator of NLRP3 inflammasome activation and the eventual formation of activated caspase 1 [93]. Preventing the interaction of TXNIP with NLRP3 will, therefore, have positive effects by reversing or restraining AD pathology [94,111]. Another idea that supports the TXNIP link to AD is glucose control and metabolism associated with neurodegeneration [93]. Although insulin-like metabolic deformities associated with Aβ functions are vague, however, a hypothesized term diabetes type-3 has been suggested recently, for integrated cerebral diabetes, categorizing insulin resistance as independent and overlapping in a few onsets of diabetes with ultimate lack of neuronal response to insulin-related signaling and a decrease in glucose metabolism [112]. Coequal clinical studies confirm that T2DM positive data remained significantly associated with the neuropathology of AD in the presence of ApoE ε4-allele carrier-patients [113]. Epidemiological data validation confirms that insulin-resistant patients are prone to AD-associated dementia and that antidiabetic medication was effective in reducing or reversing risk factors in AD [114]. Recent studies suggest that T2DM (neurovascular-disorder) has not shown any significant correlation with associated biomarkers in mild cognitive disorders in AD, and PD (neurodegenerative-disorders) pathologies [115], although the common biomarkers they tested for reference disorders do not include TXNIP which can be studied in this context. Conversely, it is also suggested that both diseases significantly correlate at early onsets of AD-symptoms [115]. At present, it is an emerging concern since anti-diabetic Food and Drug Administration approved insulin-sensitive drugs are showing positive effects on dementia risk factors via blocking TXNIP expression downstream associated with inflammatory signaling [116,117,118].

#### 3.2.4. Parkinson’s Disease (PD)

PD is the second most common neurodegenerative disease among the elderly and includes motor symptoms such as tremors, postural instability, and bradykinesia [119]. PD is characterized by the accretion of filamentous aggregates, with alpha-synuclein (α-syn) as primary precursors, as well as dopaminergic-neuron loss [120,121]. The prevailing theory suggests that the loss of dopaminergic neurons is associated with apoptosis, autophagy, and necrosis [122,123]. Recent data uncovered pyroptosis with a release of proinflammatory cytokines including IL-1β, IL-18, and nuclear protein high mobility group box 1 [124,125]. As pyroptosis is implemented by six conserved domain pore-forming proteins; among them, GSDMD (a gasdermin) is likely cleaved by caspases 11, 4, and 5 in humans [119,126]. It is claimed that pyroptosis is primarily associated with the activation of NLRP3, which is further on upstream is integrated with TXNIP. It has also been confirmed that FOXO1 is upregulated in PD targeted by mi-RNA 135b in MPP+ treated SHSY5y and PC12 cell-lines, whereas the FOXO1–TXNIP–TXN activation cascade interactions have already been confirmed from the perspective of TXNIP regulation [127,128,129]. Additionally, the majority of data highlight the participation of microRNAs and other mediators in PD pathology [130,131]. Recently, downregulation of miR-135b was shown to have a protective effect against PD pathology via promoting FOXO1 upregulation, TXNIP-mediated NLRP3 inflammasome activation, and pyroptosis [130]. TLR4 (Toll-like receptor 4) has an explicit connection to NLRP3 in the presence of myeloid differentiation of primary response protein 88 (MyD88) [132,133]. Many studies have reported improvement in PD symptoms after prevention of NLRP3-dependent pyroptosis. Indirect control inhibits the TLR4–MyD88–NF-κB signaling cascade, thereby reducing the production of NLRP3, pro-IL-1β, and pro-IL-18. The direct approach involves suppression of the TXNIP–NLRP3–caspase 1 signaling cascade [133]. These studies suggest that inhibition of pyroptosis or administration of TXNIP may be a novel therapeutic strategy against PD through direct or indirect NLRP3 activation.

## 4. TXNIP Is a Potential Therapeutic Target

TXNIP has attracted considerable attention regarding drug development owing to its multiple functions and involvement in metabolic disorders, inflammation, neurodegenerative disorders as well as cancer. Overexpression of TXNIP can be caused by various signals, such as nutritional stimuli, glucose, amino acids, and insulin, suggesting the significance of TXNIP in the regulation of metabolic and neurodegenerative diseases [8,134,135,136]. By contrast, TXNIP being a participant of apoptosis inducer and metabolic re-programmer works as a tumor suppressor; therefore, downregulation of TXNIP contributes to cancer progression [14,15,137,138], although such anticancer functions of TXNIP are associated to apoptotic pathways [56,139]. Thus, TXNIP agonist might help in anticancer treatments, raising yet another debate. In particular, accumulated data provided strong evidence that TXNIP inhibition is a potential therapeutic approach for metabolic disorders and associated diseases [12,140]. On a cellular level under oxidative-stress the metabolic functions of TXNIP are regulated partially independent of TXN1 [141]. So far, there is no specific inhibitor for TXNIP in clinical trials. Efforts are needed to develop novel TXNIP specific inhibitors to de-intensify the pro-oxidant activities of TXNIP. Although, several in vitro and in vivo studies are underway that either antagonize TXNIP directly or block it through extracellular and intracellular signaling by means of inhibitors, such as small-molecule inhibitors, phytochemicals, and peptides (Table 1).

Several small-molecule drugs have been reported, most of which are being used or under clinical investigation for metabolic and neurological disorders. Verapamil and diltiazem, a nondihydropyridine calcium channel blocker, are used to treat hypertension and angina. It has been observed that verapamil and diltiazem suppress the expression of TXNIP and reverse the β-cell loss in diabetic mice via attenuating TXNIP’s proapoptotic effects [46,142,145]. Verapamil is in a phase II clinical trial for T1DM, where it is intended to reduce TXNIP expression, increase insulin production, and enhance β-cell mass. Furthermore, the efficacy of verapamil was confirmed in a study in which verapamil administration in diabetic subjects resulted in significantly lower level of fasting serum glucose than in the subjects without verapamil treatment [144]. Surprisingly, although verapamil shows promising effects in T1DM and at the late stage of T2DM, it does not show any effect in the early stage of T2DM. This may be the reason why verapamil does not reduce TXNIP expression in the liver, muscle, and adipose tissues and, therefore, does not affect the insulin sensitivity of these tissues [144,171].

Other drugs, such as allopurinol and quercetin, have been found to prevent the overexpression of TXNIP in the rat liver and activation of the NLRP3 inflammasome, and upregulation of sterol-regulatory element–binding protein 1c (SREBP-1c), SREBP-2, liver X receptor α (LXRα), fatty acid synthase, and ROS while downregulating PPARα [146]. Moreover, several other small-molecule drugs, for example, telmisartan [172], bakuchiol [173], vorinostat (SAHA) [147], trichostatin A (TSA) [149,174], imatinib [150], taurine [151], and troglitazone [153] can inhibit the expression of TXNIP. Thielen L.A. et al. recently identified a small-molecule inhibitor, SRI-37330, that effectively suppresses TXNIP expression in rats, mice, and human pancreatic islets. In addition, treatment with SRI-37330 reduces glucagon secretion and hepatic glucose production and reverses streptozotocin-induced diabetes [154]. Nonetheless, further studies are warranted to determine the therapeutic window for clinical trials.

Phytochemicals play a major role in the curative effects of plant-derived products on different diseases, including cancers, autoimmune diseases, and neurological and metabolic disorders. Fisetin and luteolin are natural flavonoids found in vegetables and fruits such as apples, grapes, strawberries, onions, and persimmon. Several in vivo studies have revealed that fisetin treatment of mice downregulates proinflammatory cytokines and ROS production and inactivates TXNIP/MAPK and TLR4/NF-ĸB signaling [155]. Thus, fisetin exerts beneficial effects on the antioxidant system and diabetes-related diseases as well exhibits anticancer activities and anti-inflammatory properties [156,157]. Treatment with luteolin protects podocytes from high-glucose induced apoptosis in the mouse podocyte cell 5 (MCP-5) cell line and blocks TXNIP and NLRP3 inflammasome [175]. Similarly, salidroside suppresses cell proliferation, high-glucose induced oxidative stress, and extracellular-matrix accumulation in rat glomerular mesangial cells (HBZY-1) by inhibiting the TXNIP/NLRP3 signal [159]. Alkaloids such as cepharanthine and piperine are widely used as antineoplastic, antiallergic, and anti-inflammatory agents and are known to ameliorate diabetic neuropathy [161,162], whereas piperine stimulates digestive enzymes and lowers lipid peroxidation [163].

Other phytochemicals have also shown promising effects against different diseases either in vitro or in preclinical models. Among them, metformin [152], apocynin [176], curcumin [177], and ginsenoside (compound K) [167] exert significant beneficial effects on the antioxidant system, inflammation, cancer, DM, and on many other disorders.

Peptides also contribute to inhibiting TXNIP and are useful for the prevention of several disorders (neurological and metabolic disorders). Thioredoxin-mimetic (TxM) peptides, Ac-Cys-Pro-Cys-amide (CB3), and Ac-Cys-Gly-Pro-Cys-amide (CB4), prevent ROS-related damage by inhibiting p38, MAPK, and c-Jun NH2-terminal kinase (JNK) and by preventing NF-κB nuclear translocation [168,169]. CB3-treated male leptin-receptor-deficient Zucker diabetic fatty (ZDF) rats show lower inflammation and decreased TXNIP/TBP-2 expression. By contrast, the AMPK pathway is activated, which results in the inhibition of the mTOR-p70S6K pathway. Furthermore, CB3 and CB4 induce apoptosis and reduce caspase 3 cleavage and PARP dissociation in human neuroblastoma SH-SY5Y cells. It has been suggested that these peptides may have a potential to prevent neurological disorders and DM [169]. Another peptide, TN13, derived from the TXNIP-p38 interaction motif, inhibits the TXNIP–p38 interaction and significantly revives aged hematopoietic stem cells (HSCs). This finding indicates that the interaction between TXNIP and p38 activates the regulatory mechanism of HSC aging and is a possible therapeutic target for the reactivation of aging HSCs [170].

In recent years, researchers have recognized the role of microRNAs as essential mediators in the control of gene expression via post-transcriptional regulation. Here, we discuss some microRNAs that are potentially relevant for regulating TXNIP and inflammatory diseases (Table 2). MiR-20a negatively regulates the NLRP3 inflammatory response in rheumatoid arthritis fibroblast-like synoviocytes. The overexpression of miR-20a reduces TXNIP expression and downregulates the NLRP3 inflammasome and subsequent secretion of cytokine IL-1β, caspase 1, and matrix metalloproteinase 1 (MMP-1) [178]. Furthermore, the expression of miR-23a is decreased in the blood plasma of patients with central nervous system (CNS) diseases (e.g., ischemic stroke or multiple sclerosis), it also regulates neuropathic pain [179,180]. Besides, downregulation of miR-23a increases chemokine CXC receptor 4 (CXCR4) expression in a neuropathic pain model [89].

In addition, several other microRNAs have modulatory functions in the pathogenesis of some diseases. For instance, miR-377 overexpression promotes oxidative stress and increases the production of fibronectin in diabetic neuropathy [181,182]. Under stress conditions (ERS), the levels of miR-17-5p decrease, leading to inflammasome activation and causing retinal inflammation [183,184]. In contrast, miR-148a inhibits the expression of TXNIP and prevents the activation of the NLRP3 inflammasome [129]. MiR-33 increases ROS production and regulates the activity of the NLRP3 inflammasome in chronic inflammatory diseases [185].

Major efforts are needed to develop drugs that can specifically inhibit TXNIP and are highly effective in overcoming neurological and metabolic abnormalities.

## 5. Future Prospects

This review summarizes the direct effects and potential mechanisms of action of TXNIP in several metabolic and neurodegenerative disorders. TXNIP targeting has provided considerable and unique therapeutic opportunities concerning T1DM, T2DM, and the prevention of their long-term complications by improving insulin secretion and sensitivity along with β-cell function and integrity. Other comorbidities of diabetic complications, such as multiple sclerosis, β-cell mass expansion in aged mice, and glucagon action in hepatocytes, are noteworthy. These health problems should be monitored via assays of TXNIP inhibitors in vitro to gain insights into relevant functional alterations. In addition, preclinical and clinical evidence is crucial for understanding the relevance of TXNIP-specific inhibitors for the development of new promising agents to prevent DM-associated health problems. A prospective study involving a large number of patients is needed to decipher the clinical impact of vitamin D3 on DR in association with TXNIP inhibition. Inhibition of TXNIP in DM pharmacotherapy may not be effective in people with a complete lack of TXNIP. This protein works together with inflammation and oxidative stress to manifest DN and diabetic neuropathy; although the underlying mechanisms are yet to be revealed, the available data on AD should be stratified by the distinct ages of the affected brains.

## 6. Conclusions

These data collectively reveal that TXNIP inhibition may be beneficial, if applicable to diabetic patients, as well as in brain-associated diseases such as acute brain injury, ischemic stroke, trauma, and PD. The consequences of the complete loss of this protein are elusive as most of the effects of TXNIP actions are seen through animal studies at the cellular level in several diseases only. Nonetheless, TXNIP also induces apoptosis in brain cells; therefore, persistent depletion might be harmful. Thus, designing a partial agonist and testing it at the molecular level may be more appropriate in this context.

## Figures and Tables

**Table 1:** Therapeutic modulators of TXNIP. COPD: chronic obstructive pulmonary disease; CTCL: cutaneous T-cell lymphoma; DN: diabetic nephropathy; DR: diabetic retinopathy; HSCs: hematopoietic stem cells; T1DM: type 1 diabetes mellitus; T2DM: type 2 diabetes mellitus.

Type	Compound Name	Target	Diseases and Therapeutic Effects	Status	Reference/Clinicaltrials.gov
Small-molecule drug	Verapamil	Calcium channel/TXNIP	T1DM	Phase II	[142,143,144] NCT02372253
			Diabetic cardiomyopathy	In vivo	[145]
	Diltiazem	Calcium channel/TXNIP	Diabetes	In vivo	[142]
	Allopurinol	NLRP3/TXNIP/ ROS/PPARα	Inflammation, diabetes	In vivo	[146]
	Vorinostat	TXNIP	tumors	In vivo	[147]
	Trichostatin A	HDAC/TXNIP	DR	In vivo	[148,149]
	Imatinib	ABL-IRE1α/TXNIP	Diabetes	In vivo	[150]
	Taurine	Calcium channels/TXNIP	T1DM, T2DM	Phase I/II	[151] NCT01226537
	Metformin	TXNIP	T2DM	In vivo	[152]
	Troglitazone	Trx2/Ask1	Cell injury		[153]
	SRI-37330	TXNIP	Diabetes, obesity	Preclinical	[154]
Phytochemicals	Quercetin	NLRP3, TXNIP, ROS, and PPARα	T1DM	Preclinical	[146]
	Fisetin	TXNIP/MAPKs, TLR4/NF-ĸB, and ROS	Inflammation, antioxidant, anticancer actions	In vivo	[155,156,157]
	Luteolin	TXNIP/NLRP3 inflammasome	antioxidant, inflammation	In vitro	[158]
	Salidroside	TXNIP/NLRP3	T2DM, nephropathy, neuroinflammation, antioxidant	In vivo	[159,160]
	Cepharanthine	TXNIP/NLRP3	anti-inflammatory, DN	In vivo	[161,162]
	Piperine	TXNIP/NLRP3	anti-inflammatory, DN	In vivo	[162,163]
	Apocynin	NLRP3/TXNIP	Antioxidant, anti-inflammatory, heart problems	In vitro	[164]
	Puerarin	NLRP3/TXNIP	Antioxidant, anti-inflammatory, heart problems	In vitro	[164]
	Curcumin	TXNIP	diabetic vascular inflammation	In vivo	[165]
	Ginsenoside (compound K)	TXNIP/NLRP3	antidiabetic, anti-inflammatory actions	In vitro	[166,167]
Peptides	CB3	p38MAPK/JNK/NF-κB	Neurological diseases, diabetes, inflammation	In vivo	[168,169]
	CB4	p38^MAPK^/JNK/NF-κB	Neurological diseases, diabetes, inflammation	In vivo	[168,169]
	TN13	TXNIP-p38	Affects aging of HSCs	In vivo	[170]

**Table 2:** The miRNAs that regulate TXNIP. ALD: alcoholic liver disease; RA FLS: rheumatoid arthritis fibroblast-like synoviocytes.

miRNAs	Molecular Target	Type of Disease	Molecular Mechanisms	Reference
miR-20a	TXNIP	RA FLS	Downregulation of TXNIP expression; Downregulation of NLRP3, ASC and caspase-1	[178]
miR-23a	CXCR4	Neuropathic pain, multiple sclerosis	Inhibition of CXCR4; Downregulation of the TXNIP/NLRP3 inflammasome	[89,179,180]
miR-377	Not defined	DN, kidney podocyte injury	Increased fibronectin production in diabetic nephropathy; Activation of the p38 MAPK/TXNIP pathway; Upregulation of the NLRP3 inflammasome	[181,182]
miR-17-5p	TXNIP	Retinal inflammation, hypoxia-ischemia	Instability of TXNIP mRNA; Downregulation of the NLRP3 inflammasome	[183,184]
miR-148a	TXNIP	ALD	Reduction of pyroptosis; Downregulation of the NLRP3 inflammasome	[129]

## Data Availability

Not applicable

## Abbreviations

α-syn	Alpha-synuclein
AD	Alzheimer’s disease
AMPK	AMP-dependent-protein kinase
ASK-1	Apoptosis signal-regulating kinase-1
Aβ	Amyloid-β
CXCR4	Chemokine CXC receptor 4
DM	Diabetes mellitus
DN	Diabetic nephropathy
DR	Diabetic retinopathy
EBI	Early brain injury
ERS	Endoplasmic-reticulum-stress
FOXO1	Forkhead Box O1
Glut1	Glucose transporter 1
GSDMD	Gasdermin
HDAC2	Histone deacetylase 2
HIFs	Hypoxia-induced transcription factors
MAPK	Mitogen-activated protein kinase
MMP-1	Matrix metalloproteinase-1
mTOR	Mammalian target of rapamycin
NLRP3	NOD-, LRR- and pyrin domain-containing protein 3
NO	Nitric oxide
PD	Parkinson’s disease
PERK	PKR-like ER-resistant kinase
PPAR-1α	Peroxisome proliferator-activated receptors-1α
PTEN	Phosphatase and tensin homolog
ROS	Reactive oxygen species
SAH	Subarachnoid hemorrhage
T1DM	Type 1 diabetes mellitus
T2DM	Type 2 diabetes mellitus
TCA	Tricarboxylic acid cycle
TXN	Thioredoxin
TxM	Thioredoxin mimetic
TXNIP	Thioredoxin-interacting protein
VCAM-1	Vascular cell adhesion molecule-1

## References

[1] Advani A., Gilbert R.E., Thai K., Gow R.M., Langham R.G., Cox A.J., Connelly K.A., Zhang Y., Herzenberg A.M., Christensen P.K. (2009). Expression, localization, and function of the thioredoxin system in diabetic nephropathy. J. Am. Soc. Nephrol..

[2] Alhawiti N.M., Al Mahri S., Aziz M.A., Malik S.S., Mohammad S. (2017). TXNIP in Metabolic Regulation: Physiological Role and Therapeutic Outlook. Curr. Drug Targets.

[3] Yamawaki H., Pan S., Lee R.T., Berk B.C. (2005). Fluid shear stress inhibits vascular inflammation by decreasing thioredoxin-interacting protein in endothelial cells. J. Clin. Investig..

[4] Spindel O.N., World C., Berk B.C. (2012). Thioredoxin interacting protein: Redox dependent and independent regulatory mechanisms. Antioxid. Redox Signal..

[5] Tinkov A.A., Bjorklund G., Skalny A.V., Holmgren A., Skalnaya M.G., Chirumbolo S., Aaseth J. (2018). The role of the thioredoxin/thioredoxin reductase system in the metabolic syndrome: Towards a possible prognostic marker?. Cell. Mol. Life Sci..

[6] Lu J., Holmgren A. (2014). The thioredoxin antioxidant system. Free Radic. Biol. Med..

[7] Ji Cho M., Yoon S.J., Kim W., Park J., Lee J., Park J.G., Cho Y.L., Hun Kim J., Jang H., Park Y.J. (2019). Oxidative stress-mediated TXNIP loss causes RPE dysfunction. Exp. Mol. Med..

[8] Parikh H., Carlsson E., Chutkow W.A., Johansson L.E., Storgaard H., Poulsen P., Saxena R., Ladd C., Schulze P.C., Mazzini M.J. (2007). TXNIP regulates peripheral glucose metabolism in humans. PLoS Med..

[9] Hu J., Yu Y. (2018). The Function of Thioredoxin-Binding Protein-2 (TBP-2) in Different Diseases. Oxid Med. Cell. Longev..

[10] Omar D.F., Kamal M.M., El-Hefnawy M.H., El-Mesallamy H.O. (2018). Serum Vitamin D and Its Upregulated Protein, Thioredoxin Interacting Protein, Are Associated With Beta-Cell Dysfunction in Adult Patients With Type 1 and Type 2 Diabetes. Can. J. Diabetes.

[11] Li N., Zhou H., Wu H., Wu Q., Duan M., Deng W., Tang Q. (2019). STING-IRF3 contributes to lipopolysaccharide-induced cardiac dysfunction, inflammation, apoptosis and pyroptosis by activating NLRP3. Redox Biol..

[12] Nasoohi S., Ismael S., Ishrat T. (2018). Thioredoxin-Interacting Protein (TXNIP) in Cerebrovascular and Neurodegenerative Diseases: Regulation and Implication. Mol. Neurobiol..

[13] Kim H., Park H.J., Choi H., Chang Y., Park H., Shin J., Kim J., Lengner C.J., Lee Y.K., Kim J. (2019). Modeling G2019S-LRRK2 Sporadic Parkinson’s Disease in 3D Midbrain Organoids. Stem Cell Rep..

[14] Xie M., Xie R., Xie S., Wu Y., Wang W., Li X., Xu Y., Liu B., Zhou Y., Wang T. (2020). Thioredoxin interacting protein (TXNIP) acts as a tumor suppressor in human prostate cancer. Cell Biol. Int..

[15] Chen Y., Ning J., Cao W., Wang S., Du T., Jiang J., Feng X., Zhang B. (2020). Research Progress of TXNIP as a Tumor Suppressor Gene Participating in the Metabolic Reprogramming and Oxidative Stress of Cancer Cells in Various Cancers. Front. Oncol..

[16] Cadenas C., Franckenstein D., Schmidt M., Gehrmann M., Hermes M., Geppert B., Schormann W., Maccoux L.J., Schug M., Schumann A. (2010). Role of thioredoxin reductase 1 and thioredoxin interacting protein in prognosis of breast cancer. Breast Cancer Res..

[17] Ginsburg M., Maclusky N.J., Morris I.D., Thomas P.J. (1977). The specificity of oestrogen receptor in brain, pituitary and uterus. Br. J. Pharmacol..

[18] Le Jan S., Le Meur N., Cazes A., Philippe J., Le Cunff M., Leger J., Corvol P., Germain S. (2006). Characterization of the expression of the hypoxia-induced genes neuritin, TXNIP and IGFBP3 in cancer. FEBS Lett..

[19] Schulze P.C., Liu H., Choe E., Yoshioka J., Shalev A., Bloch K.D., Lee R.T. (2006). Nitric oxide-dependent suppression of thioredoxin-interacting protein expression enhances thioredoxin activity. Arterioscler. Thromb. Vasc. Biol..

[20] Wang Y., De Keulenaer G.W., Lee R.T. (2002). Vitamin D(3)-up-regulated protein-1 is a stress-responsive gene that regulates cardiomyocyte viability through interaction with thioredoxin. J. Biol. Chem..

[21] Shaked M., Ketzinel-Gilad M., Ariav Y., Cerasi E., Kaiser N., Leibowitz G. (2009). Insulin counteracts glucotoxic effects by suppressing thioredoxin-interacting protein production in INS-1E beta cells and in *Psammomys obesus* pancreatic islets. Diabetologia.

[22] Muoio D.M. (2007). TXNIP links redox circuitry to glucose control. Cell Metab..

[23] Li X., Kover K.L., Heruth D.P., Watkins D.J., Moore W.V., Jackson K., Zang M., Clements M.A., Yan Y. (2015). New Insight into Metformin Action: Regulation of ChREBP and FOXO1 Activities in Endothelial Cells. Mol. Endocrinol..

[24] Matthews J.R., Wakasugi N., Virelizier J.L., Yodoi J., Hay R.T. (1992). Thioredoxin regulates the DNA binding activity of NF-kappa B by reduction of a disulphide bond involving cysteine 62. Nucleic Acids Res..

[25] Okamoto T., Ogiwara H., Hayashi T., Mitsui A., Kawabe T., Yodoi J. (1992). Human thioredoxin/adult T cell leukemia-derived factor activates the enhancer binding protein of human immunodeficiency virus type 1 by thiol redox control mechanism. Int. Immunol..

[26] Xu G., Chen J., Jing G., Shalev A. (2013). Thioredoxin-interacting protein regulates insulin transcription through microRNA-204. Nat. Med..

[27] Abderrazak A., Syrovets T., Couchie D., El Hadri K., Friguet B., Simmet T., Rouis M. (2015). NLRP3 inflammasome: From a danger signal sensor to a regulatory node of oxidative stress and inflammatory diseases. Redox Biol..

[28] Pirzada R.H., Javaid N., Choi S. (2020). The Roles of the NLRP3 Inflammasome in Neurodegenerative and Metabolic Diseases and in Relevant Advanced Therapeutic Interventions. Genes (Basel).

[29] Zhou R., Tardivel A., Thorens B., Choi I., Tschopp J. (2010). Thioredoxin-interacting protein links oxidative stress to inflammasome activation. Nat. Immunol..

[30] Donath M.Y., Dalmas E., Sauter N.S., Boni-Schnetzler M. (2013). Inflammation in obesity and diabetes: Islet dysfunction and therapeutic opportunity. Cell Metab..

[31] Ke R., Wang Y., Hong S., Xiao L. (2020). Endoplasmic reticulum stress related factor IRE1alpha regulates TXNIP/NLRP3-mediated pyroptosis in diabetic nephropathy. Exp. Cell Res..

[32] Zhang J., Tian X., Yin H., Xiao S., Yi S., Zhang Y., Zeng F. (2020). TXNIP induced by MondoA, rather than ChREBP, suppresses cervical cancer cell proliferation, migration and invasion. J. Biochem..

[33] Gao C., Wang R., Li B., Guo Y., Yin T., Xia Y., Zhang F., Lian K., Liu Y., Wang H. (2020). TXNIP/Redd1 signalling and excessive autophagy: A novel mechanism of myocardial ischaemia/reperfusion injury in mice. Cardiovasc. Res..

[34] Feng Y.J., Wang J., Cao Z.J., Li D., Huo H.Y., Zhang X.M., Jiao X.Y. (2018). Angiotensin II promotes the expression of TXNIP through angiotensin II type 1 receptor in islet beta cells. Sheng Li Xue Bao.

[35] Kibbe C., Chen J., Xu G., Jing G., Shalev A. (2013). FOXO1 competes with carbohydrate response element-binding protein (ChREBP) and inhibits thioredoxin-interacting protein (TXNIP) transcription in pancreatic beta cells. J. Biol. Chem..

[36] Amin F.M., Abdelaziz R.R., Hamed M.F., Nader M.A., Shehatou G.S.G. (2020). Dimethyl fumarate ameliorates diabetes-associated vascular complications through ROS-TXNIP-NLRP3 inflammasome pathway. Life Sci..

[37] Park S.J., Kim J.M., Kim J., Hur J., Park S., Kim K., Shin H.J., Chwae Y.J. (2018). Molecular mechanisms of biogenesis of apoptotic exosome-like vesicles and their roles as damage-associated molecular patterns. Proc. Natl. Acad. Sci. USA.

[38] Zhou R., Yazdi A.S., Menu P., Tschopp J. (2011). A role for mitochondria in NLRP3 inflammasome activation. Nature.

[39] Blouet C., Schwartz G.J. (2011). Nutrient-sensing hypothalamic TXNIP links nutrient excess to energy imbalance in mice. J. Neurosci..

[40] Chaturvedi R.K., Flint Beal M. (2013). Mitochondrial diseases of the brain. Free Radic. Biol. Med..

[41] Shin D., Jeon J.H., Jeong M., Suh H.W., Kim S., Kim H.C., Moon O.S., Kim Y.S., Chung J.W., Yoon S.R. (2008). VDUP1 mediates nuclear export of HIF1alpha via CRM1-dependent pathway. Biochim. Biophys. Acta.

[42] Saxena G., Chen J., Shalev A. (2010). Intracellular shuttling and mitochondrial function of thioredoxin-interacting protein. J. Biol. Chem..

[43] Hui S.T., Andres A.M., Miller A.K., Spann N.J., Potter D.W., Post N.M., Chen A.Z., Sachithanantham S., Jung D.Y., Kim J.K. (2008). Txnip balances metabolic and growth signaling via PTEN disulfide reduction. Proc. Natl. Acad. Sci. USA.

[44] Duncan J.G., Fong J.L., Medeiros D.M., Finck B.N., Kelly D.P. (2007). Insulin-resistant heart exhibits a mitochondrial biogenic response driven by the peroxisome proliferator-activated receptor-alpha/PGC-1alpha gene regulatory pathway. Circulation.

[45] Ren X., Ma H., Qiu Y., Liu B., Qi H., Li Z., Kong H., Kong L. (2015). The downregulation of thioredoxin accelerated Neuro2a cell apoptosis induced by advanced glycation end product via activating several pathways. Neurochem. Int..

[46] Kim G.S., Jung J.E., Narasimhan P., Sakata H., Chan P.H. (2012). Induction of thioredoxin-interacting protein is mediated by oxidative stress, calcium, and glucose after brain injury in mice. Neurobiol. Dis..

[47] Yoshihara E., Masaki S., Matsuo Y., Chen Z., Tian H., Yodoi J. (2014). Thioredoxin/Txnip: Redoxisome, as a redox switch for the pathogenesis of diseases. Front. Immunol..

[48] Ishrat T., Mohamed I.N., Pillai B., Soliman S., Fouda A.Y., Ergul A., El-Remessy A.B., Fagan S.C. (2015). Thioredoxin-interacting protein: A novel target for neuroprotection in experimental thromboembolic stroke in mice. Mol. Neurobiol..

[49] Ye X., Zuo D., Yu L., Zhang L., Tang J., Cui C., Bao L., Zan K., Zhang Z., Yang X. (2017). ROS/TXNIP pathway contributes to thrombin induced NLRP3 inflammasome activation and cell apoptosis in microglia. Biochem. Biophys. Res. Commun..

[50] Zhao Q., Che X., Zhang H., Tan G., Liu L., Jiang D., Zhao J., Xiang X., Sun X., He Z. (2017). Thioredoxin-Interacting Protein Mediates Apoptosis in Early Brain Injury after Subarachnoid Haemorrhage. Int. J. Mol. Sci..

[51] Choe J.Y., Kim S.K. (2017). Quercetin and Ascorbic Acid Suppress Fructose-Induced NLRP3 Inflammasome Activation by Blocking Intracellular Shuttling of TXNIP in Human Macrophage Cell Lines. Inflammation.

[52] Singh L.P., Devi T.S., Yumnamcha T. (2017). The Role of Txnip in Mitophagy Dysregulation and Inflammasome Activation in Diabetic Retinopathy: A New Perspective. JOJ Ophthalmol..

[53] Saitoh M., Nishitoh H., Fujii M., Takeda K., Tobiume K., Sawada Y., Kawabata M., Miyazono K., Ichijo H. (1998). Mammalian thioredoxin is a direct inhibitor of apoptosis signal-regulating kinase (ASK) 1. EMBO J..

[54] Sun H.Y., Hu Y.J., Zhao X.Y., Zhong Y., Zeng L.L., Chen X.B., Yuan J., Wu J., Sun Y., Kong W. (2015). Age-related changes in mitochondrial antioxidant enzyme Trx2 and TXNIP-Trx2-ASK1 signal pathways in the auditory cortex of a mimetic aging rat model: Changes to Trx2 in the auditory cortex. FEBS J..

[55] Xu L., Lin X., Guan M., Zeng Y., Liu Y. (2019). Verapamil Attenuated Prediabetic Neuropathy in High-Fat Diet-Fed Mice through Inhibiting TXNIP-Mediated Apoptosis and Inflammation. Oxid. Med. Cell. Longev..

[56] Wei M., Jiao D., Han D., Wu J., Wei F., Zheng G., Guo Z., Xi W., Yang F., Xie P. (2017). Knockdown of RNF2 induces cell cycle arrest and apoptosis in prostate cancer cells through the upregulation of TXNIP. Oncotarget.

[57] Shalev A. (2014). Minireview: Thioredoxin-interacting protein: Regulation and function in the pancreatic beta-cell. Mol. Endocrinol..

[58] Wondafrash D.Z., Nire’a A.T., Tafere G.G., Desta D.M., Berhe D.A., Zewdie K.A. (2020). Thioredoxin-Interacting Protein as a Novel Potential Therapeutic Target in Diabetes Mellitus and Its Underlying Complications. Diabetes Metab. Syndr. Obes..

[59] Rorsman P., Braun M. (2013). Regulation of insulin secretion in human pancreatic islets. Annu. Rev. Physiol..

[60] Haedersdal S., Lund A., Knop F.K., Vilsboll T. (2018). The Role of Glucagon in the Pathophysiology and Treatment of Type 2 Diabetes. Mayo Clin. Proc..

[61] Marroqui L., Alonso-Magdalena P., Merino B., Fuentes E., Nadal A., Quesada I. (2014). Nutrient regulation of glucagon secretion: Involvement in metabolism and diabetes. Nutr. Res. Rev..

[62] Wilde B.R., Ye Z., Lim T.Y., Ayer D.E. (2019). Cellular acidosis triggers human MondoA transcriptional activity by driving mitochondrial ATP production. Elife.

[63] Nakamura J., Kamiya H., Haneda M., Inagaki N., Tanizawa Y., Araki E., Ueki K., Nakayama T. (2017). Causes of death in Japanese patients with diabetes based on the results of a survey of 45,708 cases during 2001–2010: Report of the Committee on Causes of Death in Diabetes Mellitus. J. Diabetes Investig..

[64] Adamopoulos C., Farmaki E., Spilioti E., Kiaris H., Piperi C., Papavassiliou A.G. (2014). Advanced glycation end-products induce endoplasmic reticulum stress in human aortic endothelial cells. Clin. Chem. Lab. Med..

[65] Yamagishi S., Imaizumi T. (2005). Diabetic vascular complications: Pathophysiology, biochemical basis and potential therapeutic strategy. Curr. Pharm. Des..

[66] Rains J.L., Jain S.K. (2011). Oxidative stress, insulin signaling, and diabetes. Free Radic. Biol. Med..

[67] Schulze P.C., De Keulenaer G.W., Yoshioka J., Kassik K.A., Lee R.T. (2002). Vitamin D3-upregulated protein-1 (VDUP-1) regulates redox-dependent vascular smooth muscle cell proliferation through interaction with thioredoxin. Circ. Res..

[68] Li X., Kover K.L., Heruth D.P., Watkins D.J., Guo Y., Moore W.V., He L.G., Zang M., Clements M.A., Yan Y. (2017). Thioredoxin-interacting protein promotes high-glucose-induced macrovascular endothelial dysfunction. Biochem. Biophys. Res. Commun..

[69] Dunn L.L., Simpson P.J., Prosser H.C., Lecce L., Yuen G.S., Buckle A., Sieveking D.P., Vanags L.Z., Lim P.R., Chow R.W. (2014). A critical role for thioredoxin-interacting protein in diabetes-related impairment of angiogenesis. Diabetes.

[70] Huang P.P., Fu J., Liu L.H., Wu K.F., Liu H.X., Qi B.M., Liu Y., Qi B.L. (2020). Honokiol antagonizes doxorubicininduced cardiomyocyte senescence by inhibiting TXNIPmediated NLRP3 inflammasome activation. Int. J. Mol. Med..

[71] Park Y.J., Yoon S.J., Suh H.W., Kim D.O., Park J.R., Jung H., Kim T.D., Yoon S.R., Min J.K., Na H.J. (2013). TXNIP deficiency exacerbates endotoxic shock via the induction of excessive nitric oxide synthesis. PLoS Pathog..

[72] Sun X., Jiao X., Ma Y., Liu Y., Zhang L., He Y., Chen Y. (2016). Trimethylamine N-oxide induces inflammation and endothelial dysfunction in human umbilical vein endothelial cells via activating ROS-TXNIP-NLRP3 inflammasome. Biochem. Biophys. Res. Commun..

[73] Zeng C., Wang R., Tan H. (2019). Role of Pyroptosis in Cardiovascular Diseases and its Therapeutic Implications. Int. J. Biol. Sci..

[74] Vandanmagsar B., Youm Y.H., Ravussin A., Galgani J.E., Stadler K., Mynatt R.L., Ravussin E., Stephens J.M., Dixit V.D. (2011). The NLRP3 inflammasome instigates obesity-induced inflammation and insulin resistance. Nat. Med..

[75] Ram C., Jha A.K., Ghosh A., Gairola S., Syed A.M., Murty U.S., Naidu V.G.M., Sahu B.D. (2020). Targeting NLRP3 inflammasome as a promising approach for treatment of diabetic nephropathy: Preclinical evidences with therapeutic approaches. Eur. J. Pharmacol..

[76] Funk S.D., Lin M.H., Miner J.H. (2018). Alport syndrome and Pierson syndrome: Diseases of the glomerular basement membrane. Matrix Biol..

[77] Qiu Y.Y., Tang L.Q. (2016). Roles of the NLRP3 inflammasome in the pathogenesis of diabetic nephropathy. Pharmacol. Res..

[78] Elsherbiny N.M., Al-Gayyar M.M. (2016). The role of IL-18 in type 1 diabetic nephropathy: The problem and future treatment. Cytokine.

[79] Kanasaki K., Taduri G., Koya D. (2013). Diabetic nephropathy: The role of inflammation in fibroblast activation and kidney fibrosis. Front. Endocrinol. (Lausanne).

[80] Kobayashi T., Uehara S., Ikeda T., Itadani H., Kotani H. (2003). Vitamin D3 up-regulated protein-1 regulates collagen expression in mesangial cells. Kidney Int..

[81] Han Y., Xu X., Tang C., Gao P., Chen X., Xiong X., Yang M., Yang S., Zhu X., Yuan S. (2018). Reactive oxygen species promote tubular injury in diabetic nephropathy: The role of the mitochondrial ros-txnip-nlrp3 biological axis. Redox Biol..

[82] Song S., Qiu D., Wang Y., Wei J., Wu H., Wu M., Wang S., Zhou X., Shi Y., Duan H. (2020). TXNIP deficiency mitigates podocyte apoptosis via restraining the activation of mTOR or p38 MAPK signaling in diabetic nephropathy. Exp. Cell Res..

[83] Shah A., Xia L., Masson E.A., Gui C., Momen A., Shikatani E.A., Husain M., Quaggin S., John R., Fantus I.G. (2015). Thioredoxin-Interacting Protein Deficiency Protects against Diabetic Nephropathy. J. Am. Soc. Nephrol..

[84] Singh L.P. (2013). Thioredoxin Interacting Protein (TXNIP) and Pathogenesis of Diabetic Retinopathy. J. Clin. Exp. Ophthalmol..

[85] Lu L., Lu Q., Chen W., Li J., Li C., Zheng Z. (2018). Vitamin D3 Protects against Diabetic Retinopathy by Inhibiting High-Glucose-Induced Activation of the ROS/TXNIP/NLRP3 Inflammasome Pathway. J. Diabetes Res..

[86] Miao J., Zhou X., Ji T., Chen G. (2020). NF-kappaB p65-dependent transcriptional regulation of histone deacetylase 2 contributes to the chronic constriction injury-induced neuropathic pain via the microRNA-183/TXNIP/NLRP3 axis. J. Neuroinflamm..

[87] Ashburner B.P., Westerheide S.D., Baldwin A.S. (2001). The p65 (RelA) subunit of NF-kappaB interacts with the histone deacetylase (HDAC) corepressors HDAC1 and HDAC2 to negatively regulate gene expression. Mol. Cell. Biol..

[88] Ganesh Yerra V., Negi G., Sharma S.S., Kumar A. (2013). Potential therapeutic effects of the simultaneous targeting of the Nrf2 and NF-kappaB pathways in diabetic neuropathy. Redox Biol..

[89] Pan Z., Shan Q., Gu P., Wang X.M., Tai L.W., Sun M., Luo X., Sun L., Cheung C.W. (2018). miRNA-23a/CXCR4 regulates neuropathic pain via directly targeting TXNIP/NLRP3 inflammasome axis. J. Neuroinflamm..

[90] Li L., Ismael S., Nasoohi S., Sakata K., Liao F.F., McDonald M.P., Ishrat T. (2019). Thioredoxin-Interacting Protein (TXNIP) Associated NLRP3 Inflammasome Activation in Human Alzheimer’s Disease Brain. J. Alzheimers Dis..

[91] Wang B.F., Yoshioka J. (2017). The Emerging Role of Thioredoxin-Interacting Protein in Myocardial Ischemia/Reperfusion Injury. J. Cardiovasc. Pharmacol. Ther..

[92] Pan Q., Guo K., Li Y., Tu Q. (2019). Role of TXNIP-mediated oxidative stress in delaying Alzheimer’s disease by estrogen. Zhong Nan Da Xue Xue Bao Yi Xue Ban.

[93] Nasoohi S., Parveen K., Ishrat T. (2018). Metabolic Syndrome, Brain Insulin Resistance, and Alzheimer’s Disease: Thioredoxin Interacting Protein (TXNIP) and Inflammasome as Core Amplifiers. J. Alzheimers Dis..

[94] Wang C.Y., Xu Y., Wang X., Guo C., Wang T., Wang Z.Y. (2019). Dl-3-n-Butylphthalide Inhibits NLRP3 Inflammasome and Mitigates Alzheimer’s-Like Pathology via Nrf2-TXNIP-TrX Axis. Antioxid. Redox Signal..

[95] Zhou T., Wang S., Lu K., Yin C. (2020). Long Non-Coding RNA SNHG7 Alleviates Oxygen and Glucose Deprivation/Reoxygenation-Induced Neuronal Injury by Modulating miR-9/SIRT1 Axis in PC12 Cells: Potential Role in Ischemic Stroke. Neuropsychiatr. Dis. Treat..

[96] Cao G., Jiang N., Hu Y., Zhang Y., Wang G., Yin M., Ma X., Zhou K., Qi J., Yu B. (2016). Ruscogenin Attenuates Cerebral Ischemia-Induced Blood-Brain Barrier Dysfunction by Suppressing TXNIP/NLRP3 Inflammasome Activation and the MAPK Pathway. Int. J. Mol. Sci..

[97] Baker A.F., Koh M.Y., Williams R.R., James B., Wang H., Tate W.R., Gallegos A., Von Hoff D.D., Han H., Powis G. (2008). Identification of thioredoxin-interacting protein 1 as a hypoxia-inducible factor 1alpha-induced gene in pancreatic cancer. Pancreas.

[98] Li Y., Miao L.Y., Xiao Y.L., Huang M., Yu M., Meng K., Cai H.R. (2015). Hypoxia induced high expression of thioredoxin interacting protein (TXNIP) in non-small cell lung cancer and its prognostic effect. Asian Pac. J. Cancer Prev..

[99] Fan J., Lv H., Li J., Che Y., Xu B., Tao Z., Jiang W. (2019). Roles of Nrf2/HO-1 and HIF-1alpha/VEGF in lung tissue injury and repair following cerebral ischemia/reperfusion injury. J. Cell. Physiol..

[100] Wang G.L., Jiang B.H., Rue E.A., Semenza G.L. (1995). Hypoxia-inducible factor 1 is a basic-helix-loop-helix-PAS heterodimer regulated by cellular O2 tension. Proc. Natl. Acad. Sci. USA.

[101] Gorgens S.W., Benninghoff T., Eckardt K., Springer C., Chadt A., Melior A., Wefers J., Cramer A., Jensen J., Birkeland K.I. (2017). Hypoxia in Combination With Muscle Contraction Improves Insulin Action and Glucose Metabolism in Human Skeletal Muscle via the HIF-1alpha Pathway. Diabetes.

[102] Liang Y., Che X., Zhao Q., Darwazeh R., Zhang H., Jiang D., Zhao J., Xiang X., Qin W., Liu L. (2019). Thioredoxin-interacting protein mediates mitochondrion-dependent apoptosis in early brain injury after subarachnoid hemorrhage. Mol. Cell. Biochem..

[103] Li Y., Li J., Li S., Li Y., Wang X., Liu B., Fu Q., Ma S. (2015). Curcumin attenuates glutamate neurotoxicity in the hippocampus by suppression of ER stress-associated TXNIP/NLRP3 inflammasome activation in a manner dependent on AMPK. Toxicol. Appl. Pharmacol..

[104] Zhao Q., Che X., Zhang H., Fan P., Tan G., Liu L., Jiang D., Zhao J., Xiang X., Liang Y. (2017). Thioredoxin-interacting protein links endoplasmic reticulum stress to inflammatory brain injury and apoptosis after subarachnoid haemorrhage. J. Neuroinflamm..

[105] Kaya B., Erdi F., Kilinc I., Keskin F., Feyzioglu B., Esen H., Karatas Y., Uyar M., Kalkan E. (2015). Alterations of the thioredoxin system during subarachnoid hemorrhage-induced cerebral vasospasm. Acta Neurochir..

[106] Xu W., Li T., Gao L., Zheng J., Yan J., Zhang J., Shao A. (2019). Apelin-13/APJ system attenuates early brain injury via suppression of endoplasmic reticulum stress-associated TXNIP/NLRP3 inflammasome activation and oxidative stress in a AMPK-dependent manner after subarachnoid hemorrhage in rats. J. Neuroinflamm..

[107] Xu W., Yan J., Chen S., Ocak U., Shao A., Zhang J. (2020). Peroxisomal Dysfunction Contributes to White Matter Injury Following Subarachnoid Hemorrhage in Rats via Thioredoxin-Interacting Protein-Dependent Manner. Front. Cell Dev. Biol..

[108] Wang Y., Wang Y., Bharti V., Zhou H., Hoi V., Tan H., Wu Z., Nagakannan P., Eftekharpour E., Wang J.F. (2019). Upregulation of Thioredoxin-Interacting Protein in Brain of Amyloid-beta Protein Precursor/Presenilin 1 Transgenic Mice and Amyloid-beta Treated Neuronal Cells. J. Alzheimers Dis..

[109] Nakanishi A., Kaneko N., Takeda H., Sawasaki T., Morikawa S., Zhou W., Kurata M., Yamamoto T., Akbar S.M.F., Zako T. (2018). Amyloid beta directly interacts with NLRP3 to initiate inflammasome activation: Identification of an intrinsic NLRP3 ligand in a cell-free system. Inflamm. Regen..

[110] Tsubaki H., Tooyama I., Walker D.G. (2020). Thioredoxin-Interacting Protein (TXNIP) with Focus on Brain and Neurodegenerative Diseases. Int. J. Mol. Sci..

[111] Pan Q., Guo K., Xue M., Tu Q. (2020). Estrogen protects neuroblastoma cell from amyloid-beta 42 (Abeta42)-induced apoptosis via TXNIP/TRX axis and AMPK signaling. Neurochem. Int..

[112] De la Monte S.M. (2019). The Full Spectrum of Alzheimer’s Disease Is Rooted in Metabolic Derangements That Drive Type 3 Diabetes. Adv. Exp. Med. Biol..

[113] Malek-Ahmadi M., Beach T., Obradov A., Sue L., Belden C., Davis K., Walker D.G., Lue L., Adem A., Sabbagh M.N. (2013). Increased Alzheimer’s disease neuropathology is associated with type 2 diabetes and ApoE epsilon.4 carrier status. Curr. Alzheimer Res..

[114] Bendlin B.B. (2019). Antidiabetic therapies and Alzheimer disease. Dialogues Clin. Neurosci..

[115] Gasecka A., Siwik D., Gajewska M., Jaguszewski M.J., Mazurek T., Filipiak K.J., Postula M., Eyileten C. (2020). Early Biomarkers of Neurodegenerative and Neurovascular Disorders in Diabetes. J. Clin. Med..

[116] Tang G., Duan F., Li W., Wang Y., Zeng C., Hu J., Li H., Zhang X., Chen Y., Tan H. (2019). Metformin inhibited Nod-like receptor protein 3 inflammasomes activation and suppressed diabetes-accelerated atherosclerosis in apoE(-/-) mice. Biomed. Pharmacother..

[117] Chaudhari K., Reynolds C.D., Yang S.H. (2020). Metformin and cognition from the perspectives of sex, age, and disease. Geroscience.

[118] Rotermund C., Machetanz G., Fitzgerald J.C. (2018). The Therapeutic Potential of Metformin in Neurodegenerative Diseases. Front. Endocrinol..

[119] Zhang X., Zhang Y., Li R., Zhu L., Fu B., Yan T. (2020). Salidroside ameliorates Parkinson’s disease by inhibiting NLRP3-dependent pyroptosis. Aging.

[120] Gasser T. (2009). Molecular pathogenesis of Parkinson disease: Insights from genetic studies. Expert Rev. Mol. Med..

[121] Schapira A.H., Bezard E., Brotchie J., Calon F., Collingridge G.L., Ferger B., Hengerer B., Hirsch E., Jenner P., Le Novere N. (2006). Novel pharmacological targets for the treatment of Parkinson’s disease. Nat. Rev. Drug Discov..

[122] Liu J., Liu W., Lu Y., Tian H., Duan C., Lu L., Gao G., Wu X., Wang X., Yang H. (2018). Piperlongumine restores the balance of autophagy and apoptosis by increasing BCL2 phosphorylation in rotenone-induced Parkinson disease models. Autophagy.

[123] Ghavami S., Shojaei S., Yeganeh B., Ande S.R., Jangamreddy J.R., Mehrpour M., Christoffersson J., Chaabane W., Moghadam A.R., Kashani H.H. (2014). Autophagy and apoptosis dysfunction in neurodegenerative disorders. Prog. Neurobiol..

[124] Chen X., He W.T., Hu L., Li J., Fang Y., Wang X., Xu X., Wang Z., Huang K., Han J. (2016). Pyroptosis is driven by non-selective gasdermin-D pore and its morphology is different from MLKL channel-mediated necroptosis. Cell Res..

[125] Vande Walle L., Lamkanfi M. (2016). Pyroptosis. Curr. Biol..

[126] Rathinam V.A.K., Zhao Y., Shao F. (2019). Innate immunity to intracellular LPS. Nat. Immunol..

[127] Dumitriu A., Latourelle J.C., Hadzi T.C., Pankratz N., Garza D., Miller J.P., Vance J.M., Foroud T., Beach T.G., Myers R.H. (2012). Gene expression profiles in Parkinson disease prefrontal cortex implicate FOXO1 and genes under its transcriptional regulation. PLoS Genet..

[128] Ji L., Wang Q., Huang F., An T., Guo F., Zhao Y., Liu Y., He Y., Song Y., Qin G. (2019). FOXO1 Overexpression Attenuates Tubulointerstitial Fibrosis and Apoptosis in Diabetic Kidneys by Ameliorating Oxidative Injury via TXNIP-TRX. Oxid. Med. Cell. Longev..

[129] Heo M.J., Kim T.H., You J.S., Blaya D., Sancho-Bru P., Kim S.G. (2019). Alcohol dysregulates miR-148a in hepatocytes through FoxO1, facilitating pyroptosis via TXNIP overexpression. Gut.

[130] Zeng R., Luo D.X., Li H.P., Zhang Q.S., Lei S.S., Chen J.H. (2019). MicroRNA-135b alleviates MPP(+)-mediated Parkinson’s disease in in vitro model through suppressing FoxO1-induced NLRP3 inflammasome and pyroptosis. J. Clin. Neurosci..

[131] Niranjan R. (2018). Recent advances in the mechanisms of neuroinflammation and their roles in neurodegeneration. Neurochem. Int..

[132] Su Q., Li L., Sun Y., Yang H., Ye Z., Zhao J. (2018). Effects of the TLR4/Myd88/NF-kappaB Signaling Pathway on NLRP3 Inflammasome in Coronary Microembolization-Induced Myocardial Injury. Cell. Physiol. Biochem..

[133] Patra M.C., Shah M., Choi S. (2020). Toll-like receptor-induced cytokines as immunotherapeutic targets in cancers and autoimmune diseases. Semin. Cancer Biol..

[134] Hui T.Y., Sheth S.S., Diffley J.M., Potter D.W., Lusis A.J., Attie A.D., Davis R.A. (2004). Mice lacking thioredoxin-interacting protein provide evidence linking cellular redox state to appropriate response to nutritional signals. J. Biol. Chem..

[135] Fang S., Jin Y., Zheng H., Yan J., Cui Y., Bi H., Jia H., Zhang H., Wang Y., Na L. (2011). High glucose condition upregulated Txnip expression level in rat mesangial cells through ROS/MEK/MAPK pathway. Mol. Cell. Biochem..

[136] Shaked M., Ketzinel-Gilad M., Cerasi E., Kaiser N., Leibowitz G. (2011). AMP-activated protein kinase (AMPK) mediates nutrient regulation of thioredoxin-interacting protein (TXNIP) in pancreatic beta-cells. PLoS ONE.

[137] Jia J.J., Geng W.S., Wang Z.Q., Chen L., Zeng X.S. (2019). The role of thioredoxin system in cancer: Strategy for cancer therapy. Cancer Chemother. Pharmacol..

[138] Zhou J., Chng W.J. (2013). Roles of thioredoxin binding protein (TXNIP) in oxidative stress, apoptosis and cancer. Mitochondrion.

[139] Wang J., Wang J., Wang J.J., Zhang W.F., Jiao X.Y. (2017). Role of autophagy in TXNIP overexpression-induced apoptosis of INS-1 islet cells. Sheng Li Xue Bao.

[140] Thielen L., Shalev A. (2018). Diabetes pathogenic mechanisms and potential new therapies based upon a novel target called TXNIP. Curr. Opin. Endocrinol. Diabetes Obes..

[141] Ogata F.T., Batista W.L., Sartori A., Gesteira T.F., Masutani H., Arai R.J., Yodoi J., Stern A., Monteiro H.P. (2013). Nitrosative/oxidative stress conditions regulate thioredoxin-interacting protein (TXNIP) expression and thioredoxin-1 (TRX-1) nuclear localization. PLoS ONE.

[142] Xu G., Chen J., Jing G., Shalev A. (2012). Preventing beta-cell loss and diabetes with calcium channel blockers. Diabetes.

[143] Tfelt-Hansen P.C., Jensen R.H. (2012). Management of cluster headache. CNS Drugs.

[144] Khodneva Y., Shalev A., Frank S.J., Carson A.P., Safford M.M. (2016). Calcium channel blocker use is associated with lower fasting serum glucose among adults with diabetes from the REGARDS study. Diabetes Res. Clin. Pract..

[145] Chen J., Cha-Molstad H., Szabo A., Shalev A. (2009). Diabetes induces and calcium channel blockers prevent cardiac expression of proapoptotic thioredoxin-interacting protein. Am. J. Physiol. Endocrinol. Metab..

[146] Wang W., Wang C., Ding X.Q., Pan Y., Gu T.T., Wang M.X., Liu Y.L., Wang F.M., Wang S.J., Kong L.D. (2013). Quercetin and allopurinol reduce liver thioredoxin-interacting protein to alleviate inflammation and lipid accumulation in diabetic rats. Br. J. Pharmacol..

[147] Malone C.F., Emerson C., Ingraham R., Barbosa W., Guerra S., Yoon H., Liu L.L., Michor F., Haigis M., Macleod K.F. (2017). mTOR and HDAC Inhibitors Converge on the TXNIP/Thioredoxin Pathway to Cause Catastrophic Oxidative Stress and Regression of RAS-Driven Tumors. Cancer Discov..

[148] Drummond D.C., Noble C.O., Kirpotin D.B., Guo Z., Scott G.K., Benz C.C. (2005). Clinical development of histone deacetylase inhibitors as anticancer agents. Annu. Rev. Pharmacol. Toxicol..

[149] Perrone L., Devi T.S., Hosoya K.I., Terasaki T., Singh L.P. (2010). Inhibition of TXNIP expression in vivo blocks early pathologies of diabetic retinopathy. Cell Death Dis..

[150] Morita S., Villalta S.A., Feldman H.C., Register A.C., Rosenthal W., Hoffmann-Petersen I.T., Mehdizadeh M., Ghosh R., Wang L., Colon-Negron K. (2017). Targeting ABL-IRE1alpha Signaling Spares ER-Stressed Pancreatic beta Cells to Reverse Autoimmune Diabetes. Cell Metab..

[151] Gondo Y., Satsu H., Ishimoto Y., Iwamoto T., Shimizu M. (2012). Effect of taurine on mRNA expression of thioredoxin interacting protein in Caco-2 cells. Biochem. Biophys. Res. Commun..

[152] Chai T.F., Hong S.Y., He H., Zheng L., Hagen T., Luo Y., Yu F.X. (2012). A potential mechanism of metformin-mediated regulation of glucose homeostasis: Inhibition of Thioredoxin-interacting protein (Txnip) gene expression. Cell Signal..

[153] Lim P.L., Liu J., Go M.L., Boelsterli U.A. (2008). The mitochondrial superoxide/thioredoxin-2/Ask1 signaling pathway is critically involved in troglitazone-induced cell injury to human hepatocytes. Toxicol. Sci..

[154] Thielen L.A., Chen J., Jing G., Moukha-Chafiq O., Xu G., Jo S., Grayson T.B., Lu B., Li P., Augelli-Szafran C.E. (2020). Identification of an Anti-diabetic, Orally Available Small Molecule that Regulates TXNIP Expression and Glucagon Action. Cell Metab..

[155] Li P., Chen D., Huang Y. (2018). Fisetin administration improves LPS-induced acute otitis media in mouse in vivo. Int. J. Mol. Med..

[156] Adhami V.M., Syed D.N., Khan N., Mukhtar H. (2012). Dietary flavonoid fisetin: A novel dual inhibitor of PI3K/Akt and mTOR for prostate cancer management. Biochem. Pharmacol..

[157] Sahu B.D., Kumar J.M., Sistla R. (2016). Fisetin, a dietary flavonoid, ameliorates experimental colitis in mice: Relevance of NF-kappaB signaling. J. Nutr. Biochem..

[158] Wu J., Xu X., Li Y., Kou J., Huang F., Liu B., Liu K. (2014). Quercetin, luteolin and epigallocatechin gallate alleviate TXNIP and NLRP3-mediated inflammation and apoptosis with regulation of AMPK in endothelial cells. Eur. J. Pharmacol..

[159] Wang S., Zhao X., Yang S., Chen B., Shi J. (2017). Salidroside alleviates high glucose-induced oxidative stress and extracellular matrix accumulation in rat glomerular mesangial cells by the TXNIP-NLRP3 inflammasome pathway. Chem. Biol. Interact..

[160] Ni G.L., Cui R., Shao A.M., Wu Z.M. (2017). Salidroside Ameliorates Diabetic Neuropathic Pain in Rats by Inhibiting Neuroinflammation. J. Mol. Neurosci..

[161] Kudo K., Hagiwara S., Hasegawa A., Kusaka J., Koga H., Noguchi T. (2011). Cepharanthine exerts anti-inflammatory effects via NF-kappaB inhibition in a LPS-induced rat model of systemic inflammation. J. Surg. Res..

[162] Samra Y.A., Said H.S., Elsherbiny N.M., Liou G.I., El-Shishtawy M.M., Eissa L.A. (2016). Cepharanthine and Piperine ameliorate diabetic nephropathy in rats: Role of NF-kappaB and NLRP3 inflammasome. Life Sci..

[163] Srinivasan K. (2007). Black pepper and its pungent principle-piperine: A review of diverse physiological effects. Crit. Rev. Food Sci. Nutr..

[164] Lian D., Yuan H., Yin X., Wu Y., He R., Huang Y., Chen Y. (2019). Puerarin inhibits hyperglycemia-induced inter-endothelial junction through suppressing endothelial Nlrp3 inflammasome activation via ROS-dependent oxidative pathway. Phytomedicine.

[165] Wongeakin N., Bhattarakosol P., Patumraj S. (2014). Molecular mechanisms of curcumin on diabetes-induced endothelial dysfunctions: Txnip, ICAM-1, and NOX2 expressions. Biomed. Res. Int..

[166] Yang X.D., Yang Y.Y., Ouyang D.S., Yang G.P. (2015). A review of biotransformation and pharmacology of ginsenoside compound K. Fitoterapia.

[167] Chen W., Wang J., Luo Y., Wang T., Li X., Li A., Li J., Liu K., Liu B. (2016). Ginsenoside Rb1 and compound K improve insulin signaling and inhibit ER stress-associated NLRP3 inflammasome activation in adipose tissue. J. Ginseng Res..

[168] Kim S.R., Lee K.S., Park S.J., Min K.H., Lee M.H., Lee K.A., Bartov O., Atlas D., Lee Y.C. (2011). A novel dithiol amide CB3 attenuates allergic airway disease through negative regulation of p38 mitogen-activated protein kinase. Am. J. Respir. Crit. Care Med..

[169] Cohen-Kutner M., Khomsky L., Trus M., Ben-Yehuda H., Lenhard J.M., Liang Y., Martin T., Atlas D. (2014). Thioredoxin-mimetic peptide CB3 lowers MAPKinase activity in the Zucker rat brain. Redox Biol..

[170] Jung H., Kim D.O., Byun J.E., Kim W.S., Kim M.J., Song H.Y., Kim Y.K., Kang D.K., Park Y.J., Kim T.D. (2016). Thioredoxin-interacting protein regulates haematopoietic stem cell ageing and rejuvenation by inhibiting p38 kinase activity. Nat. Commun.

[171] Cha-Molstad H., Xu G., Chen J., Jing G., Young M.E., Chatham J.C., Shalev A. (2012). Calcium channel blockers act through nuclear factor Y to control transcription of key cardiac genes. Mol. Pharmacol..

[172] Erdi F., Keskin F., Esen H., Kaya B., Feyzioglu B., Kilinc I., Karatas Y., Cuce G., Kalkan E. (2016). Telmisartan ameliorates oxidative stress and subarachnoid haemorrhage-induced cerebral vasospasm. Neurol. Res..

[173] Liu H., Guo W., Guo H., Zhao L., Yue L., Li X., Feng D., Luo J., Wu X., Cui W. (2020). Bakuchiol Attenuates Oxidative Stress and Neuron Damage by Regulating Trx1/TXNIP and the Phosphorylation of AMPK After Subarachnoid Hemorrhage in Mice. Front. Pharmacol..

[174] De Marinis Y., Cai M., Bompada P., Atac D., Kotova O., Johansson M.E., Garcia-Vaz E., Gomez M.F., Laakso M., Groop L. (2016). Epigenetic regulation of the thioredoxin-interacting protein (TXNIP) gene by hyperglycemia in kidney. Kidney Int..

[175] Yu Q., Zhang M., Qian L., Wen D., Wu G. (2019). Luteolin attenuates high glucose-induced podocyte injury via suppressing NLRP3 inflammasome pathway. Life Sci..

[176] Van den Worm E., Beukelman C.J., Van den Berg A.J., Kroes B.H., Labadie R.P., Van Dijk H. (2001). Effects of methoxylation of apocynin and analogs on the inhibition of reactive oxygen species production by stimulated human neutrophils. Eur. J. Pharmacol..

[177] Witkin J.M., Li X. (2013). Curcumin, an active constiuent of the ancient medicinal herb *Curcuma longa* L.: Some uses and the establishment and biological basis of medical efficacy. CNS Neurol. Disord. Drug Targets.

[178] Li X.F., Shen W.W., Sun Y.Y., Li W.X., Sun Z.H., Liu Y.H., Zhang L., Huang C., Meng X.M., Li J. (2016). MicroRNA-20a negatively regulates expression of NLRP3-inflammasome by targeting TXNIP in adjuvant-induced arthritis fibroblast-like synoviocytes. Joint Bone Spine.

[179] Fenoglio C., Ridolfi E., Cantoni C., De Riz M., Bonsi R., Serpente M., Villa C., Pietroboni A.M., Naismith R.T., Alvarez E. (2013). Decreased circulating miRNA levels in patients with primary progressive multiple sclerosis. Mult. Scler..

[180] Jia L., Hao F., Wang W., Qu Y. (2015). Circulating miR-145 is associated with plasma high-sensitivity C-reactive protein in acute ischemic stroke patients. Cell Biochem. Funct..

[181] Wang Q., Wang Y., Minto A.W., Wang J., Shi Q., Li X., Quigg R.J. (2008). MicroRNA-377 is up-regulated and can lead to increased fibronectin production in diabetic nephropathy. FASEB J..

[182] Wang W., Ding X.Q., Gu T.T., Song L., Li J.M., Xue Q.C., Kong L.D. (2015). Pterostilbene and allopurinol reduce fructose-induced podocyte oxidative stress and inflammation via microRNA-377. Free Radic. Biol. Med..

[183] Lerner A.G., Upton J.P., Praveen P.V., Ghosh R., Nakagawa Y., Igbaria A., Shen S., Nguyen V., Backes B.J., Heiman M. (2012). IRE1alpha induces thioredoxin-interacting protein to activate the NLRP3 inflammasome and promote programmed cell death under irremediable ER stress. Cell Metab..

[184] Coucha M., Mohamed I.N., Elshaer S.L., Mbata O., Bartasis M.L., El-Remessy A.B. (2017). High fat diet dysregulates microRNA-17-5p and triggers retinal inflammation: Role of endoplasmic-reticulum-stress. World J. Diabetes.

[185] Xie Q., Wei M., Zhang B., Kang X., Liu D., Zheng W., Pan X., Quan Y., Liao D., Shen J. (2018). MicroRNA33 regulates the NLRP3 inflammasome signaling pathway in macrophages. Mol. Med. Rep..

